# Immediate effect of physical activity on gut motility in healthy adults

**DOI:** 10.1038/s41598-025-18860-8

**Published:** 2025-09-29

**Authors:** Kento Katagiri, Soichiro Koyama, Kotaro Takeda, Kouji Yamada, Koki Tan, Hikaru Kondo, Yohei Otaka, Shigeo Tanabe

**Affiliations:** 1 Department of Rehabilitation Medicine, School of Medicine, Fujita Health University, Toyoake, Japan; 2 Graduate School of Health Sciences, Fujita Health University, Toyoake, Japan; 3 Research Center for Robotic Smart Home and Activity Assistive Technology, Fujita Health University, Toyoake, Japan

**Keywords:** Bowel sound, Intestinal sound, Aerobic exercise, Constipation, Intestinal peristalsis, Health care, Therapeutics

## Abstract

Constipation is a prevalent condition that negatively impacts health and quality of life. Inadequate physical activity is a known contributing factor, often associated with reduced gut motility. However, the physiological mechanism linking physical activity and constipation remains unclear. Particularly research on the immediate effects of physical activity on peristalsis is scarce. Therefore, we aimed to elucidate this mechanism by examining the immediate effects of physical activity on gut motility in healthy adults. Twenty-one participants were instructed to walk on a treadmill for 20 min. Bowel sounds were assessed at rest and at intervals up to 15 min after walking. Bowel sounds were used as indirect markers of gut motility. We calculated the sum of the absolute signal amplitudes of bowel sounds, the percentage of bowel sounds duration, and number of discrete bowel sounds, which have been proposed as indices of gut motility. All the indices increased significantly 1–2 min post-exercise compared to resting values. This increase may be attributed to changes in the autonomic nervous system and local reflexes caused by biomechanical oscillations. In addition, gut motility activation might explain the effects of physical activity intervention on constipation and offer insights into its potential role in managing the condition.

## Introduction

Constipation is a gastrointestinal condition frequently encountered by healthcare professionals in clinical practice, affecting 0.7–29.6% of people^1^. Constipation is characterized by symptoms related to difficulties in defecation^2^ and can lead to sleep disorders^3^, anxiety, depression, and other psychological problems. It can also increase the risk of mortality associated with hypertension, cardiovascular diseases, and cerebrovascular diseases, as well as the incidence of colon cancer^4,5^. In addition, constipation is associated with the life prognosis and quality of life^6,7^, making it an important medical issue that necessitates management.

The effects of physical activity on constipation have been well-investigated, with insufficient physical activity and excessive sedentary behavior being associated with constipation^8^. Compared to weekly activity, daily physical activity is associated with a lower likelihood of developing constipation^9^. Moreover, individuals walking less than 0.5 km daily have an increased risk of constipation^10^. While these findings suggest an association between physical activity and constipation, the mechanism by which physical activity affects constipation remains unknown.

Patients with constipation have been reported to exhibit reduced gut motility, including decreased peristaltic movement^11–13^. Peristalsis is defined as a gastrointestinal motor pattern that moves luminal contents in the anal direction^14^. Therefore, an increase in the peristaltic movement may be beneficial for relieving constipation.

Although methods such as endoscopy and magnetic resonance imaging (MRI) can be used to assess gut motility, these techniques are often invasive and costly, which makes them impractical for real-time and repeated measurements in healthy individuals. Bowel sounds [BSs] have been used as indirect measurements of gut motility since the early 1900s when their analysis was first reported by Cannon^15^. The BS is produced by the turbulence caused by gut motility during the movement of liquids and gases through the intestinal tract^16–19^. Therefore, BS is considered to objectively reflect gut motility in real-time^20^. In recent years, advances in recording devices, including wireless stethoscopes, have facilitated the exploration of BS analyses from various perspectives. In healthy individuals, BSs have been shown to correlate with gut motility^19,21–23^. A previous study proposed the sound index [SI]^22^, BS duration, and number of BSs as indicators^24,25^ for evaluating the immediate effects on gut motility. Moreover, the SI, which is the sum of the absolute signal amplitudes, correlates with peristaltic movement^22^. The percentage of BS duration and number of BS are used to evaluate increases and decreases in gut motility^24,25^. The normal number of BSs ranges from 5 to 35 sounds/min^26–28^.

In the present study, we aimed to clarify part of the mechanism of constipation by examining the immediate effects of walking, as an example of physical activity, on gut motility in healthy adults.

## Methods

### Study design and participants

This study employed a single-arm, pre-test/post-test design. A convenience sample of 21 healthy adults, with no history of orthopedic, gastrointestinal, neurological, respiratory, cardiovascular, or psychiatric diseases, participated in the study. The mean age of the participants was 21 years (standard deviation [SD], 0.4; 11 women). Before the experiment, the objectives and procedures of the study were explained to the participants. After that, informed consent was obtained from all participants. This study was conducted in accordance with the principles of the Declaration of Helsinki. The study protocol was approved by the Institutional Ethics Committee of Fujita Health University (approval no. HB20-294).

### Experimental setup

**Fig. 1:**
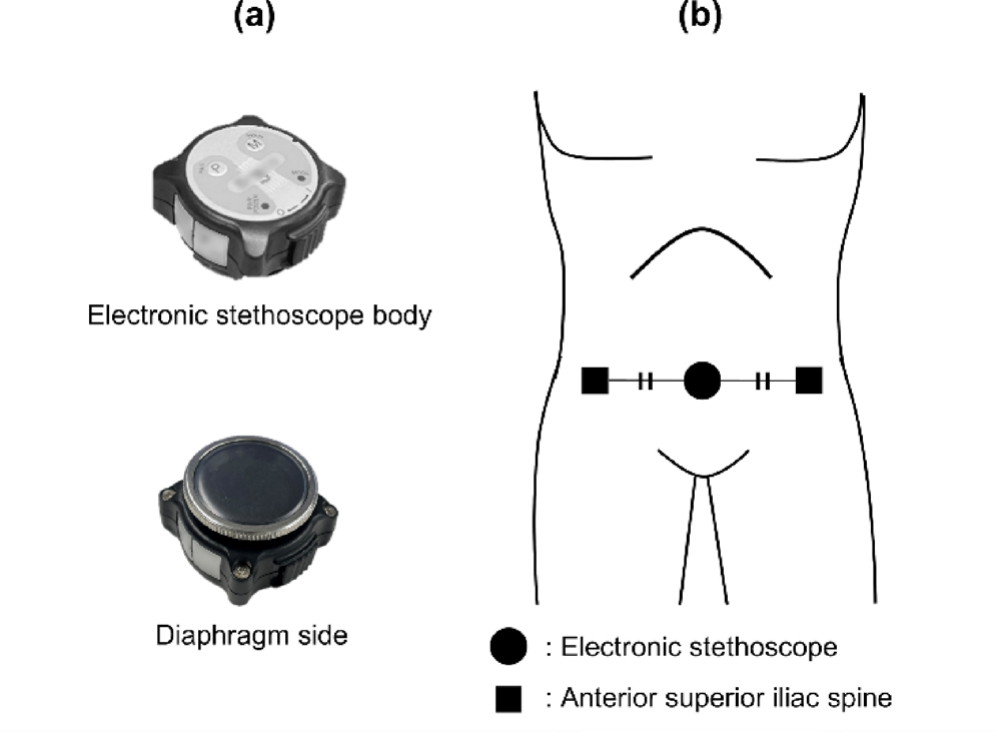
View of the stethoscope. (**a**) Appearance of the electronic stethoscope and (**b**) Mounting position of the electronic stethoscope. The electronic stethoscope was positioned at the center of the superior anterior iliac spine.

The experimental setup consisted of an electronic stethoscope (Bresco K13002-1; AD Soar Corporation, Kawasaki, Japan), a personal computer (Endeavor NA513E; Seiko Epson Corporation, Suwa, Japan), and a treadmill (Aeromill STM-1250; Nihon Kohden Corporation, Tokyo, Japan). Figure 1a depicts an external view of the electronic stethoscope.

The electronic stethoscope used in this study recorded sounds remotely via wireless communication and allowed for the selection of auditory ranges based on the purpose of each measurement. The frequency of the most common BSs ranges between 100 and 300 Hz in both healthy adults and patients with bowel obstruction^29^ and has three peak frequencies at 273, 612, and 632 Hz^30^. Therefore, broadband mode (40–800 Hz) was adopted for the measurements in this study.

Figure 1b illustrates the mounting position of the electronic stethoscope. A band made of stretchable fabric was used to secure the stethoscope at the center of the superior anterior iliac spine on both sides. To ensure close contact between the vibrating membrane of the electronic stethoscope and the skin, the electronic stethoscope was pressed against the abdomen of the participant until the skin was slightly indented.

Data measured using the electronic stethoscope were imported to a personal computer through a microphone terminal. The Windows Voice Recorder software (Microsoft Corporation, Redmond, WA, USA) was used to record data from the electronic stethoscope, with a sampling frequency of 1 kHz.

### Experimental procedure

Based on the findings of a previous study, the experiment was performed at least 2 h after each meal to exclude the effect of food intake on gut motility^31^. First, based on a previous study^32^, BSs were measured in the supine position for 1 min after a 5-min rest period to stabilize autonomic nervous system activity. The usual time required to walk 10 m at ground level was assessed to calculate the comfortable walking speed. Then, participants walked on a treadmill. As the comfortable walking speed on the treadmill is slower than on the ground level^33^, the walking speed was set at 70% of the comfortable walking speed on ground level. Given that walking at least 3000 steps improves bowel clearance^34^, the walking duration was set to 20 min, assuming the completion of 3000 steps. Subsequently, BSs were measured for 15 min, starting 1 min after the end of the walk. Silence was maintained in the measurement environment to ensure that BSs were detected without noise interference. During all BS measurements, the participants were maintained in the supine position.

### Data and statistical analysis

For the analysis, the SI, percentage of BS duration, and number of BSs were calculated. The SI was the sum of the absolute values of the amplitudes of the BSs during each 1 min. To calculate the percentage of BS duration and the number of BSs, sound waveforms were divided by 1 min, and the mean + 2SD of amplitude at each time point was calculated. Peak amplitudes exceeding the mean + 2SD at each time point were then extracted as BSs. The criteria for separating individual BSs were based on the method described in a previous study^29^. If the interval between peaks was within 0.2 s, it was defined as the same BS; if the interval was > 0.2 s, it was defined as another BS. These calculations were performed using the LabVIEW 2021 software (National Instruments, Austin, TX, USA).

Data from eight time periods were used: at rest, 1–2 min after walking, 2–3 min after walking, 3–4 min after walking, 4–5 min after walking, 5–6 min after walking, 10–11 min after walking, and 15–16 min after walking. The Shapiro–Wilk test was used to assess normality. Mean values were reported for normally distributed data, whereas median values were used for non-normally distributed data.

Comparisons were conducted between pre-intervention and post-intervention for each of the following: SI, percentage of BS duration, and number of BS. Multiple two-tailed *t*-tests or Wilcoxon signed rank tests with Bonferroni corrections for multiple comparisons were used for analysis. Statistical analyses were performed using the R software (version 4.0.3; The R Foundation for Statistical Computing, Vienna, Austria). The statistical significance level was set at *p* < 0.05.

## Results

**Fig. 2:**
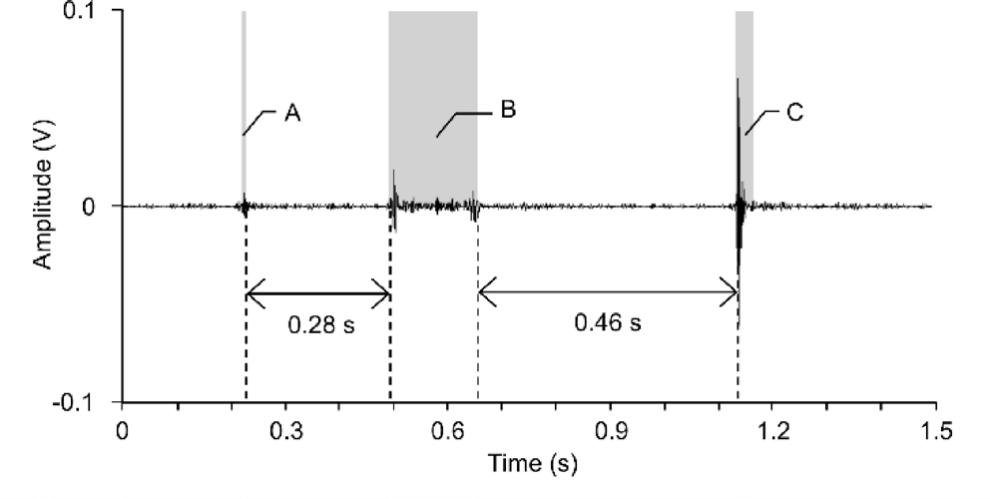
Raw data of bowel sounds recorded using an electronic stethoscope. This data presents bowel sounds of 1.5 s, not the entire measured period for one participant. Gray shading indicates the estimated bowel sound range. In this example, three bowel sounds were identified according to the defined method, as A and B, and B and C were separated by > 0.2 s and > 0.4 s, respectively.

**Fig. 3:**
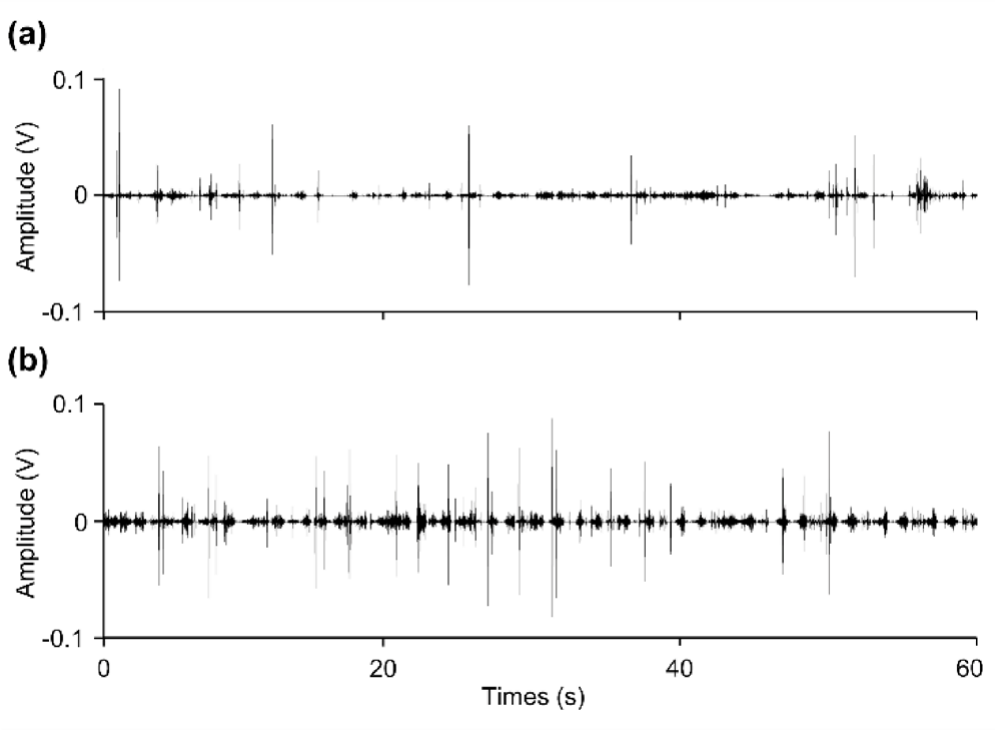
One-minute bowel sounds recorded waveforms. (**a**) One-minute bowel sounds waveform at rest and (**b**) 1–2 min after walking. This data represents an example from a single participant.

All participants completed all experimental tasks without dropping out. No adverse events were observed. Figure 2 presents BSs of 1.5 s, not the entire measured period for one participant. Gray shading indicates the estimated BS range. In this example, three BSs were determined because A and B and B and C were separated by > 0.2 s and > 0.4 s, respectively. Figure 3 shows the 1-min BS waveforms recorded in one example at rest and 1–2 min after walking (upper and lower diagrams, respectively). Compared to at rest, an increase in the amplitude and variability of the waveform was observed 1–2 min after walking.

**Fig. 4:**
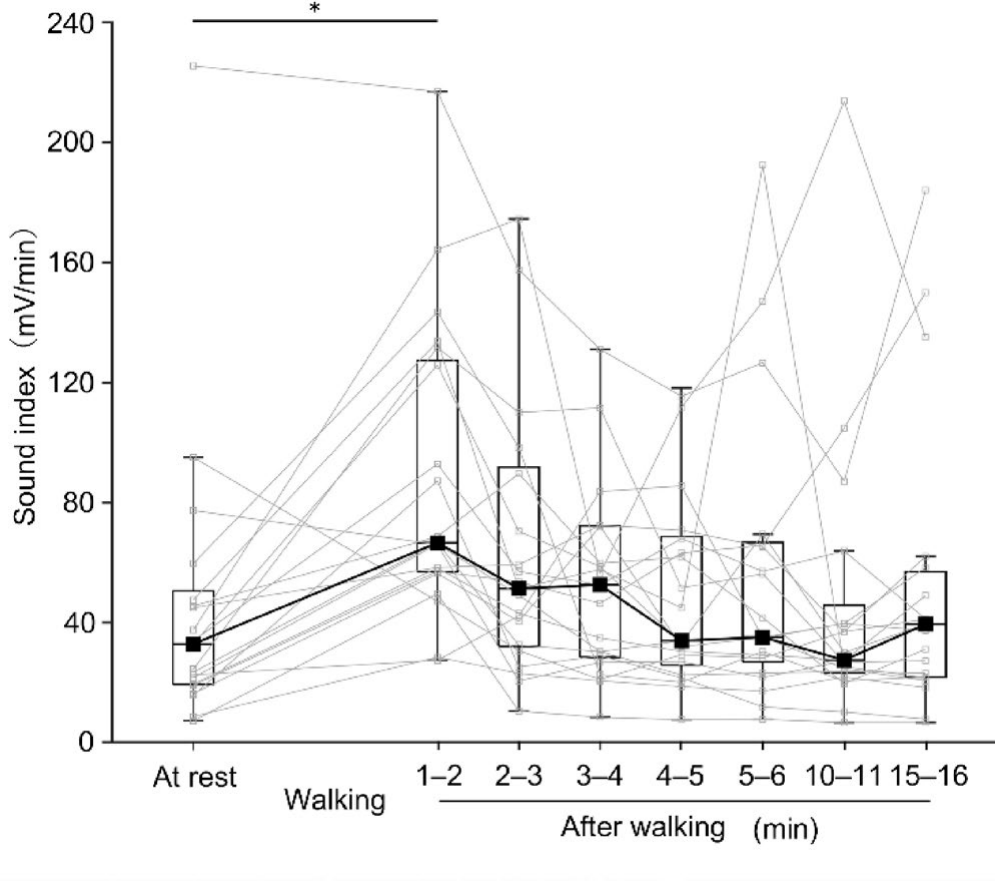
Median sound index for each evaluation period. Small squares and thin lines represent individual participant data, while large squares and thick lines represent the median. *p < 0.05.

**Fig. 5:**
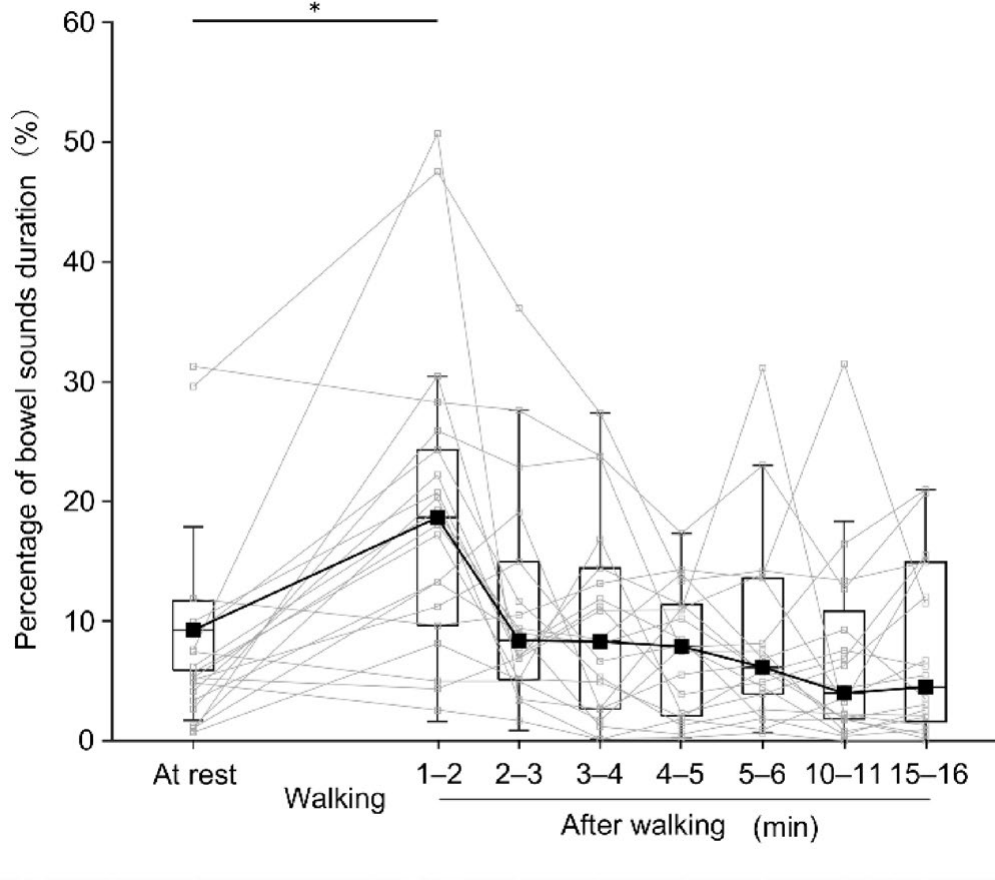
Median percentage of bowel sound duration for each evaluation period. Small squares and thin lines represent individual participant data, while large squares and thick lines represent the median. *p < 0.05.

**Fig. 6:**
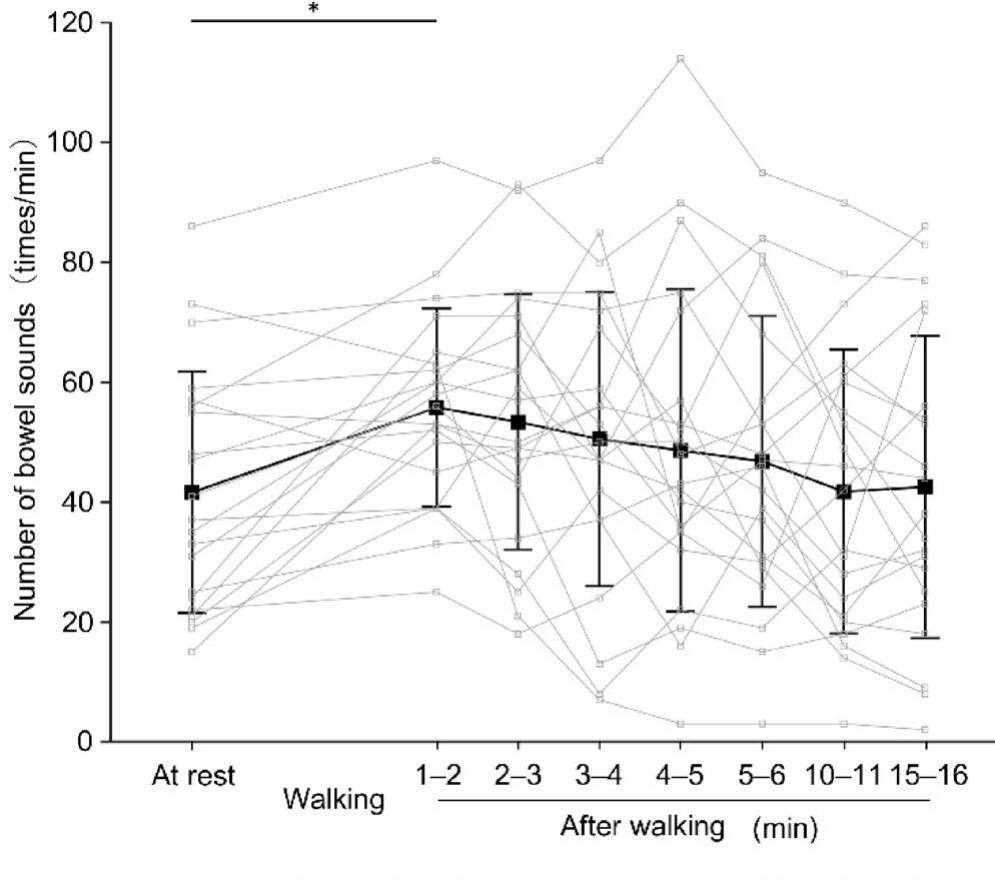
Mean number of bowel sounds for each evaluation period. Small squares and thin lines represent individual participant data, while large squares and thick lines represent the mean. *p < 0.05.

Figure 4 depicts the changes in the SI over time. The Wilcoxon signed rank test with Bonferroni corrections revealed significant differences (adjusted *p* = 0.0067) between at rest and 1–2 min after walking (32.80 mV/min [19.46–47.50] and 66.56 mV/min [57.04–125.82], respectively). Figure 5 illustrates the changes in the percentage of BS duration over time. The Wilcoxon signed rank test with Bonferroni corrections revealed significant differences (adjusted* p* = 0.0037) between at rest and 1–2 min after walking (5.32% [3.37–8.88] and 19.25% [11.21–24.30], respectively). Figure 6 presents the changes in the number of BSs over time. Paired-sample *t*-tests with Bonferroni corrections revealed significant differences (adjusted* p* = 0.022) between at rest and 1–2 min after walking (41.62 ± 19.63 times/min and 55.81 ± 16.13 times/min, respectively). Overall, for all three indicators, the values increased immediately (1–2 min) after walking compared to at rest and returned to baseline levels (at rest) within 2–3 min after walking or later.

## Discussion

This study investigated the immediate effect of physical activity on gut motility using three indicators calculated based on the BS. Walking, as a form of physical activity, increased the values of all three indicators of gut motility. Specifically, the SI, percentage of BS duration, and number of BS increased 1–2 min after walking compared to those at rest. This increase may be attributed to the changes in gut motility induced by physical activity. A previous study has reported that the SI is closely correlated to peristaltic movement^22^. The increase in the SI immediately after physical activity may have resulted from increased peristaltic movement. Moreover, the immediate increase in the percentage of BS duration and number of BSs, which are used to evaluate changes in gut motility^24^, may support the possibility that peristaltic movement increases immediately after physical activity.

The increase in gut motility immediately after physical activity may be caused by two factors: changes in the systemic autonomic nervous system and/or local reflexes caused by biomechanical oscillations.

In terms of changes in the systemic autonomic nervous system, the parasympathetic nervous system may stimulate gut motility, immediately leading to increased gut motility. Autonomic nervous system changes during exercise are commonly assessed through heart rate measurements^35,36^. During exercise, parasympathetic tone decreases and sympathetic tone increases, thereby increasing the heart rate^35,36^. Subsequently, the increase in heart rate during exercise rapidly decreases after the end of exercise—a change attributed to the vagus nerve (the parasympathetic nervous system)^37^. Moreover, the autonomic nervous system is essential for modulating visceral organ function^38^, and the extrinsic autonomic nervous system impacts gut motility indirectly by interacting with the enteric nervous system, which in turn promotes smooth muscle cell activity^39,40^. Autonomic nervous system changes may be the underlying factor for the immediate increase in gut motility; however, this could not be confirmed in this study. Future studies incorporating indicators of autonomic balance, such as heart rate variability^41^, are needed to clarify this mechanism.

In terms of changes in local reflexes caused by biomechanical oscillations, physical activity may cause biomechanical oscillations. These oscillations may trigger local reflexes and subsequently increase gut motility. As walking is a major part of physical activity^42^, we study used walking as an intervention. During one walking cycle, the center of gravity sway is approximately 23 mm vertically and 46 mm horizontally^43^. This swaying of the center of gravity may have a similar effect to biomechanical oscillations. Although walking was not used as an intervention, several studies have demonstrated the effects of whole-body vibration on constipation. These studies indicated that whole-body vibration stimulation improves constipation^44–46^. The authors also suggested that the factors that contribute to this influence are similar to the giant migratory contractions of the colon, which occur in association with the gastrocolic response. Therefore, biomechanical oscillations, such as whole-body vibration, may enhance stool passage through the descending colon to the rectum. Subsequently, the presence of feces in the rectum stimulates the local reflexes, thereby promoting gut motility.

Regarding clinical significance, patients with constipation have been reported to exhibit reduced gut motility, including decreased peristaltic movement^12,13^. Given that peristalsis is essential for transporting luminal contents through the gut tract^14^, the immediate increase in peristaltic movement observed in this study may be beneficial for relieving constipation. This short-term increase in gut motility, when repeatedly activated and summed, may be beneficial for a long-term impact on gut motility (e.g., relieving constipation). Although the precise mechanisms underlying these effects have not yet been fully elucidated, previous studies have reported that periodic interventions providing mechanical stimulation to the colon, such as vibration therapy and vibrating capsule therapy, can relieve constipation^44,47–51^. Vibration therapy involves sessions of 20 min once daily for 12 weeks or of 15 min thrice a week for two weeks^44,47^. These interventions share similarities with physical activity in providing mechanical stimulation to the gut, as well as in intervention duration and repetition frequency. However, the duration, frequency, and intensity of physical activity require further investigation to determine optimal intervention protocols. Regarding the autonomic nervous system, previous studies have shown that differences in exercise duration result in varying changes in autonomic nervous system activity after exercise cessation^52^. Further research is necessary to determine whether different durations, frequencies, or intensities of physical activity can induce more sustained changes in gut motility beyond the immediate response. Additionally, verification is needed to determine whether immediate effect of gut motility has long-term homeostatic effects.

Notably, to the best of our knowledge, this is the first study to demonstrate the immediate effects of walking on gut motility using BS analysis. This work may contribute to understanding the mechanisms by which physical activity relieves constipation. Our findings indicate that walking immediately enhances gut motility through measurable changes in BS parameters, thereby highlighting the importance of physical activity interventions in constipation management. The clinical significance of this study is that such activity can be safely performed by anyone, making it widely applicable and easy to incorporate into daily life.

It should be noted that the interpretation of BSs remains controversial. Although a previous study has reported a correlation between BSs and peristaltic movement^22^, another study suggests that BSs may also reflect segmental contractions^21^. Segmental contractions contribute to mixing and retention of intestinal contents, and when excessive, can lead to constipation^53^. Therefore, caution is required when interpreting BSs as a measure of gut motility or peristaltic movement.

However, this study has several limitations. The first is an imbalance in the male-to-female ratio and age of the participants. A previous study suggested that female hormones affect colon motility^54^. In addition, older individuals, particularly women, are more prone to constipation than men^55^. Second, the findings of this study may not be fully generalizable to patients with gastrointestinal disorders such as constipation or other digestive diseases. In such populations, gut motility is often inherently reduced or increased due to impaired motility^56,57^. In addition, the physiological response may be blunted in patient populations with impaired gut motility. For example, patients with constipation may exhibit limited response to stimulant laxatives^58^. Third, BS is an indirect assessment of gut motility; therefore, their interpretation should be approached with caution. Previous studies reported that BSs correlate with gut motility^19,21–23^. However, no significant correlation has been observed between auscultated BSs and gut motility within a given region^25^, since BSs are easily transmitted throughout the abdominal cavity^25^. Moreover, BSs are generated by gut motility (e.g., intestinal peristalsis, anti-peristalsis, saccular oscillations, segmental contractions), which promotes the movement of liquids and gases through the intestinal tract^16–18^. Given that no standard indicator has been established for gut motility using the BS, a continued exploratory study using multiple indicators calculated from BSs is needed. Fourth, in this preliminary study, BSs were recorded with participants in a supine position to ensure measurement stability. Consequently, as a factor other than walking, postural changes may have contributed to the observed effects. The transition from supine to standing may have induced physical (gravitational and intra‑abdominal pressure) and autonomic (sympathetic activation) changes, both of which could have influenced the observed BSs^59,60^. Fifth, to exclude the immediate effects of food intake on gut motility, the experiment was conducted at least 2 h after each meal. However, the participants’ habitual diet may have influenced baseline gut motility. Previous studies have reported that certain foods, such as dietary fiber, can enhance gut motility and shorten transit time^61^. Finally, participants were informed about the study objectives, which may have influenced gut motility through psychological mechanisms. Previous studies have shown that strong psychological stress can alter autonomic function and increase gut motility^62^. Although the psychological stress involved in the present study was minimal compared with that involved in previous studies, gut motility may still have been affected. Importantly, these approaches were not included in the present study. To address these limitations mentioned above, future studies should include more diverse populations, adopt both direct and indirect assessments of gut motility, incorporate methods to comprehensively evaluate physiological responses to physical activity, and consider controlling psychological factors that may influence gut motility.

## Conclusion

The present study examined the immediate effect of physical activity on gut motility using the SI, percentage of BS duration, and number of BSs. All three indicators significantly increased 1 min after physical activity, suggesting that gut motility increased at that time point. This increase may be related to changes in the systemic autonomic nervous system and local reflexes caused by biomechanical oscillations. These findings suggest that physical activity immediately increases gut motility, thereby explaining the effects of physical activity on constipation.

## Data Availability

The data that support the findings of this study are available from the corresponding author upon reasonable request.
